# Portable sterilizer with microbe content detection device

**DOI:** 10.1186/s42269-021-00496-z

**Published:** 2021-02-04

**Authors:** Sreerup Banerjee, Shriram Raghunathan, Saubhik Banerjee, Boudhayan Bandyopadhyay

**Affiliations:** 1 Department of Mechanical Engineering, Haldia Institute of Technology, Haldia, West Bengal India; 2 School of Computer Science and Engineering, VIT Bhopal University, Kothri Kalan, Madhya Pradesh India; 3 Glocal Healthcare Systems Private Limited, Kolkata, India; 4 Department of Biotechnology, School of Life Science and Biotechnology, Adamas University, Kolkata, India

**Keywords:** Sterilization, Ultraviolet, Microbe detection, Laser speckle, Image processing

## Abstract

**Background:**

Infectious diseases, such as the latest COVID pandemic, caused by microorganisms like bacteria and virus, wreak havoc shaking human civilization with its rapid infection rate, and high number of mortalities. In case of a contagious disease, the virus can survive on any surface over a period of time and can be transferred to the human host through touching those surfaces unknowingly. Cleaning those possible surfaces to which these microorganisms can cling onto is one of the major ways to curb the spread. The aim of this study was to design a sterilizer which can clean such surfaces of daily used items easily within a certain period of time and can assess the cleaning efficacy by estimating the presence of microbes before and after sanitization.

**Method development:**

To achieve this goal, we propose a portable sterilization unit that contains a sterilization chamber fitted with a microbe content detector. The sterilization chamber will cleanse the surfaces off the microbes using ultraviolet radiation. The chamber can be portable and at the same time big enough to accommodate items of daily use, like watch, wallet, clothes, utensils to even foods for single-house application. The microbe content detector will detect the success of the sterilization procedure by examining the time-lapse laser speckle images captured by a high-speed camera by mean of image processing algorithm, such that the user can determine whether further sterilization is required.

**Conclusions:**

Microbe content detection device associated with the conventional sterilization procedure will give an assessment of the effectiveness of the sterilization. Successful implementation of sterilization for a wide variety of items of everyday use aided with microbe content detection technique is first of its kind and should be an effective tool for use in large communities, offices and public places for effective sterilization to help fight against the spread of infectious diseases.

## Background

Over the centuries, different pathogenic microorganisms created havoc to the human civilization with their rapid spread and mortality rate. (Jarus 2020) Earlier world has seen a huge number of mortalities due to epidemic or pandemic caused by microorganisms that had put their scars on the face of human civilization, such as plague, cholera, different kinds of influenza, Acute Immunodeficiency Syndrome (AIDS). The present days are a testing one for humankind as another pandemic is currently sweeping the world: coronavirus disease (COVID-19) pandemic, caused by severe acute respiratory syndrome coronavirus 2 (SARS-CoV-2), commonly known as novel corona virus. With its incredible rate of infection and considerable mortality, it has shaken the normal life across the world. As per WHO report, as of 13 April 2020, there have been 17,76,867 confirmed cases of COVID-19, including 1,11,828 deaths (Coronavirus (COVID-19) 2020). Scientists and medical professionals are fighting a battle to keep the rate of infection and death under control, and the situation is complicated by its relatively unknown nature to us, that prevents the development of proper treatment modality.

As there is no proven treatment protocol as of now, and a huge number of infected patients are putting an immense pressure on the total healthcare system to bring it on the verge of collapse, the only effective way to control the pandemic is to curb the spread. To curb the spread of the pandemic, experts are focussing on: (1) physical distancing based on the fact that the viruses are laterally transmitted from person-to-person and travel short distances in the form of aerosol, (2) washing hands and keeping hands away from face region, as the virus can remain stable on different surfaces for a considerable period of time and enters the body through the mouth or nasal openings, and lastly, and (3) cleaning the possible surfaces to which the viruses can adhere for a certain time span (coronavirus disease (COVID-19) advice for the public 2020; Disinfection and Sterilization 2020; Rutala and Weber 2016). Scientific studies reported that novel coronavirus can remain stable for a considerable period of time in certain surfaces, e.g., up to 72 h on plastic and stainless steel surfaces (van Doremalen et al. 2020).

Sterilization procedure to kill or deactivate the pathogenic microorganisms (Disinfection and Sterilization 2020; Yoo 2018) from a surface is thus an effective tool to control the spread of infectious diseases including COVID-19. If it is possible to check the efficiency of sterilization process, it would act as an added factor of hygiene as it is assessing the fitness for reusing the item without risking spread or contamination of the microorganism. The aim of this study is to achieve the goal by designing a portable sterilizer unit coupled with a microbe content detection device for sterilization of the surfaces of different articles of daily use.

There are different techniques for sterilization, each with its own advantages and drawbacks. The most common and easiest of all the methods is incineration. This method is heat based and cannot be applicable for a large range of items that are to be recycled or reused. Another popular heat-based method is keeping the items to be sterilized in an autoclave by heating them up to the boiling point of water at an elevated pressure (Hugo 1991). Autoclave suffers from the common drawback of the heat-based techniques, and it can damage items which cannot withstand heat. Moreover, the machine is expensive. One of the prominent non-heating sterilization techniques involves the use of chemicals that acts as germicides. Ethylene oxide (EtO) and hydrogen peroxide vapor can act as very good sterilizing agents for items where application of heat is not suitable (Yoo 2018; Wallace 2016). These techniques have their own drawbacks owing to the toxic nature of EtO and thus cannot be used for all the items (McDonnell and Russell 1999) while sterilization with hydrogen peroxide vapor is expensive (Dancer 2014). Other techniques involve disrupting the cellular machinery of the microorganisms with electromagnetic radiation of suitable range. Ultra-violet (UV) ray is the most common of the methods and the germicidal property of UV is known since long (Council on Physical Medicine 1948; Katara et al. 2008). Shorter wavelength of UV region, referred to as UVC, is having a wavelength range of 200–280 nm. UVC radiation denatures the DNA of microorganisms, which have a high absorbance of the UV spectrum at 254 nm, and thus referred to as industry-standard (Walker et al. 2013; Summerfelt 2003). Some studies suggest that the most common bacteria and viruses can be inactivated by UV doses of 30 mJ/cm^2^ at a wavelength of 254 nm (Wedemeyer 1996). Microwave irradiation, with a frequency range of 2455 ± 30 MHz, has also shown germicidal property (Kang and Kato 2014). It is also a heat-based property and thus cannot be used for sterilization of all types of items. Infrared (IR) radiation is another heat-based technique which has shown germicidal properties and used for mainly metallic items (Mata-Portuguez et al. 2002). IR has the typical limitation of the heat-based technique that it cannot be used for all types of item. Moreover, IR does not penetrate enough in the inner lumens of complex shaped objects to sterilize them effectively. Another popular method is the use of vibration produced by ultrasound (US) waves. Higher-power US waves in the lower frequency range (20–100 kHz) have shown to for a cavitation in a liquid filled medium to cause destruction of the microbes (Piyasena et al. 2003). Sterilization systems that uses US and UV together is also used to obtain better results and utilized for disinfecting different types of food (Khandpur and Gogate 2016).

## Method development

The proposed device will consist of two parts: (1) a sterilization unit equipped with ultraviolet (UV) exposure to disinfect articles and (2) a microbe content detection device to detect the presence of microbes to check the effectiveness of the sterilization procedure.

### Proposed sterilization chamber

**Fig. 1:**
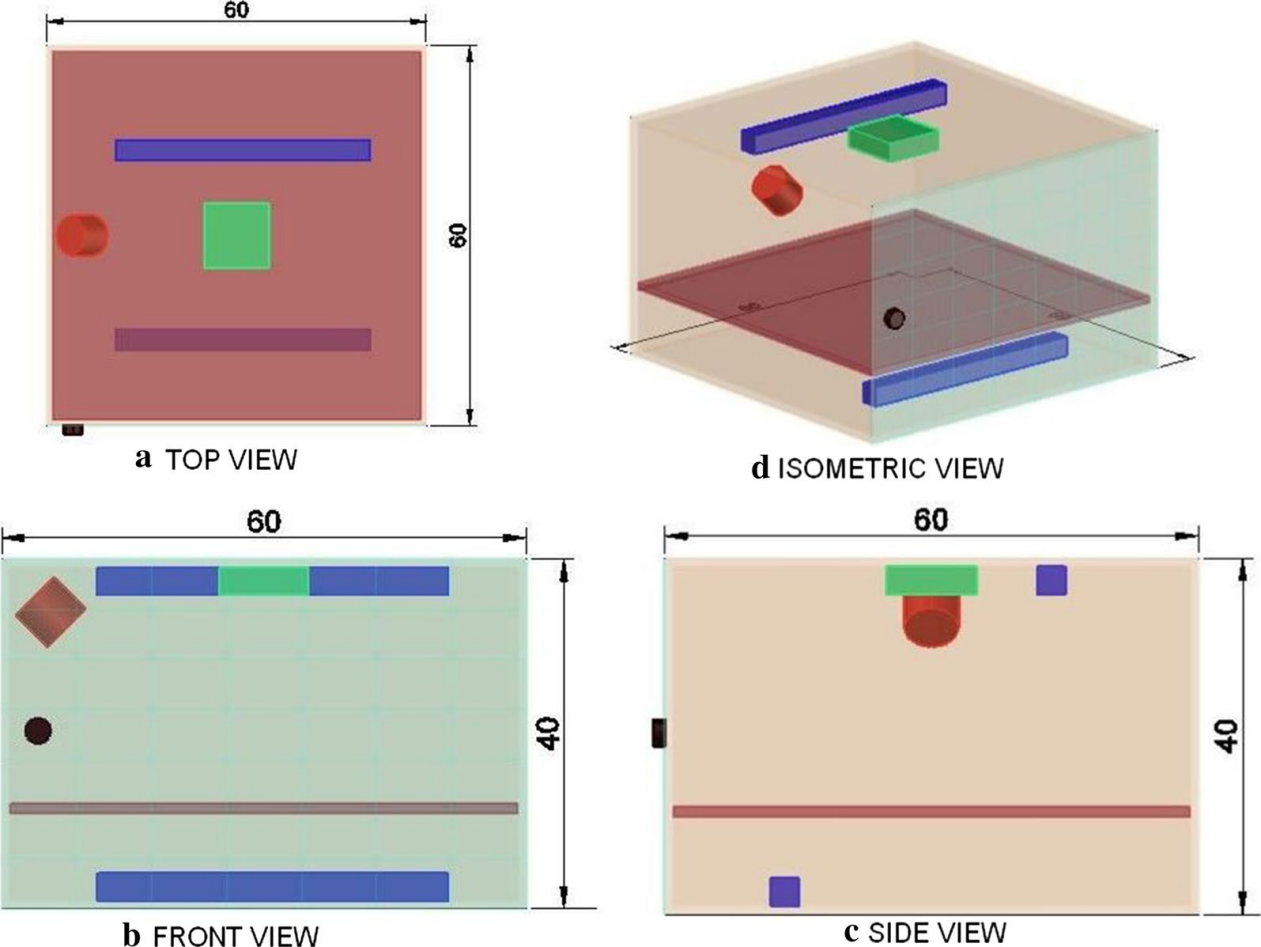
Three orthographic views (top, front, and side) and 3D (isometric) view of the schematic of the sterilization unit is depicted to show the relative positions of different components. All the measurements are in centimetres. Images not to scale. The sterilization chamber (transparent maroon box with 1 cm thickness) is made of metal with a door (transparent cyan plate with 1 cm thickness containing small black knob) hinged at the front. The laser head (red cylinder) is mounted at the top corner such that it focuses the laser at an angle of 45°. One CCD camera (green square) is mounted at the top to capture the laser speckles. The CCD camera is connected to a computational system (not shown) where the image transfer and analysis takes place. Two UV lamps (blue rectangles) are mounted at the top and bottom face of the metal chamber. A glass base plate (maroon square with 3 cm thickness) is placed on the top of the UV lamp mounted at the bottom, where the items to be sterilized will be placed. All the views of the sterilizer unit are developed using the AutoCAD version 2018 (Autodesk Inc.)

The proposed sterilization unit will have a cuboid-shaped metallic chamber with a door hinged at the front. The sterilization chamber for household application should not be very big so that it has to be kept at a stationary position, but big enough to put inside most objects of daily use, ranging from masks, mobiles, jewelleries, wallets or any wearable and even food items, for sterilization. Clothes can also be sterilized in a short period of time instead spending energy and time on washing daily. The proposed dimension of the metal chamber will be 60 × 40 × 60 cm (length–width–depth) for use in a single house. However, larger sterilization chambers can be manufactured for the version of the device meant to be used for entire community. The case will contain two ultraviolet (UV) light source, laser head and a CCD camera for microbe content detection device (detailed in the next section). The UV lamps will be placed at the top and bottom face of the chamber. A glass plate placed above the UV lamp at the bottom will be used to keep the items to be sterilized. The laser head will be mounted at the top corner of the chamber and the CCD camera will be mounted at the top face of the chamber. Schematic diagram of the sterilization chamber is presented in Fig. 1. UV light exposure for a period of 20–25 min is recommended for sterilizing the items (Katara et al. 2008). The exposure time for each item can be calibrated using the results of the microbe content detection system. The UV lamps and laser head will be electrically powered, and a battery back-up will also be there to make it effective where electric point is not readily available.

### Microbe content detection device

#### Principle of operation

The sterilizing unit can be coupled with laser-based sensor to track the effectiveness of the sterilization procedure. The principle is as follows: Once the microorganisms like bacteria, virus adhere to the surface of any object, it will try to move from one place to another, or try to reorient itself. The laser ray can monitor that dynamics through the scattering of the incident laser beam onto the object. When a red, coherent laser beam falls on the microorganism, it will be scattered by the movement of the microbes on the surface of the object. This scattering leads the light to interfere, creating a random pattern known as laser speckle. As the microbes move on the surface, the speckle pattern will change. By detecting the decorrelation in the laser speckle intensity patterns from microbes, the living activities of microorganisms can be detected. This method was reported by Yoon et al. (2016).

To monitor the change, a camera needs to be fitted inside the chamber that can record the change over a few seconds. Yoon et al. devised a system that takes images at a rate of 30 frames per second (fps) for 20 s (Yoon et al. 2016). Then the image will be processed by subtracting one from another to reveal any difference over time. That difference will give an estimation of the presence of microorganism before and after sterilization.

#### Device setup

The optical setup of the device will be made as per the protocol reported in (Yoon et al. 2016; Xu et al. 2018). The proposed design of laser speckle monitoring device is discussed in (Xu et al. 2018). Here the coherent light source is a laser operated at 633 nm wavelength. Two lenses and one pinhole will be placed to collimate the laser beam and expand its diameter according to the size of the sample to be scanned. The spot diameter on the sample surface will be 2.5 to 5 mm. The whole setup of laser beam is placed in such a way that the angle between the incident beam and the sample surface will be 45°. The scattered light from the sample will be captured by a CCD camera with a resolution of 5 × 5 pixels, which is placed in parallel to the sample surface. The lens can be fixed as per reported in (Xu et al. 2018). When the laser beam is diffracted from the sample surface, the random granular pattern will be generated which is known as laser speckle. The aperture and field depth can be adjusted to achieve the clear spackle pattern. The laser speckle pattern can be obtained taking snaps at 60 frames per second for 30 s.

#### Image processing and contamination detection

Once the time-lapse laser speckle images are ready, the images will be transferred to the analyzer unit. The analyzer unit is an embedded computational system having a microprocessor and a memory storage fitted with an interactive display. It takes the images from the camera as inputs. The input time-lapse images will then be analyzed in the computational system, based on temporal change in speckle pattern using image processing algorithm. The variation of speckle pattern over time will be traced and autocorrelation between images can be found out (Yoon et al. 2016) using the following formula:

$$C\left( {x,y;\tau } \right) = \frac{1}{T - \tau }\mathop \sum \limits_{t = 1}^{T - \tau } \overline{{I\left( {x,y;t} \right)}} \overline{{I\left( {x,y;t + \tau } \right)}} \delta t$$

where $\overline{{I\left( {x,y;t} \right)}}$ designates the image intensity over the image domain at time T. $\overline{{I\left( {x,y;t + \tau } \right)}}$ designates the image intensity over the image domain at a later time $t + \tau$ and $C\left( {x,y;\tau } \right)$ is the normalized auto-correlation coefficient that quantifies the change in speckle pattern over time. The image processing algorithm will assess the image intensity values of the input images at a time interval of τ and will determine the autocorrelation coefficient C, to give a quantitative measure of the changes in speckle pattern. If the sample surface is not contaminated, then speckle pattern will not vary over time and the algorithm will provide an autocorrelation coefficient of + 1. If the microbe is embedded onto the surface of the sample, then speckle pattern will change over time because of the movement of the microbe on the surface of the object, as shown in the proof-of-concept experiment by Yoon et al. (2016) and the autocorrelation coefficient will decrease. This variation will be the indication of presence of any microbe on the sample surface. Based on the analysis the display will show the quantitative results in terms of correlation coefficient as well as inform the user about the efficiency with which the sterilization procedure is carried out. The images can be transferred via USB port from the storage system for conducting further analysis.

## Discussion

The effectiveness of the proposal detailed in this paper to use UV sterilization technique for a given item can be assessed by the microbe content detection device. Based on the result of the microbe content detection device for the individual items, the time required for effective sterilization of individual items can be calibrated.

The analysis of the performance of the device needs to be done for different items of daily use to understand the lacunae, if any, and the challenges of the proposal. A detailed documentation of parameters to be set for a wide range of items is to be done based on the performance analysis. A real-time image analysis-based assessment of the microbe content detection device to give the feedback to the attached computational system to optimize the timing of operation of the unit for a particular class of items based on machine learning algorithm is a step in the future direction for the device set.

Even though CMOS camera has certain edge in performance over CCD camera, keeping a comparative cost-effectiveness, CCD camera is used in the proposed sterilization unit instead of CMOS camera. Despite attempts to limit the cost, the whole unit with CCD camera, and embedded computational system and display may be prohibitive for it to penetrate all the sections of the society, but its utility can balance it, and in the post-COVID pandemic attack world, a community-based approach for use of it can make it an effective tool to combat the spread of pathogenic microorganisms worldwide.

## Conclusions

The proposal detailed in this paper uses UV sterilization technique to achieve desired result for a wide variety of everyday use items, ranging from wristwatch, wallet, jewellery, clothes to food items. Microbe content detection device will give an assessment of the effectiveness of the sterilization. Such a sterilization unit, coupled with microbe content detection device, is not available for common people to the best of the knowledge for the authors and it should be an effective tool for use in large communities, offices and public places for effective sterilization.

## Data Availability

Not applicable.

## References

[CR1] Coronavirus (COVID-19) [database on the Internet]. World Health Organization. 2020. Available from: https://who.sprinklr.com/. Accessed: 13 April 2020

[CR2] Coronavirus disease (COVID-19) advice for the public [database on the Internet]. World Health Organization. 2020. Available from: https://www.who.int/emergencies/diseases/novel-coronavirus-2019/advice-for-public. Accessed: 13 April 2020

[CR3] Council on Physical Medicine (1948). J Am Med Assoc.

[CR4] Dancer SJ (2014). Controlling hospital-acquired infection: focus on the role of the environment and new technologies for decontamination. Clin Microbiol Rev.

[CR5] Disinfection and Sterilization [database on the Internet]. Centers for Disease Control and Prevention. 2020. Available from: https://www.cdc.gov/infectioncontrol/guidelines/disinfection/index.html. Accessed: 13 April 2020

[CR6] Hugo WB (1991). A brief history of heat and chemical preservation and disinfection. The Journal of Applied Bacteriology.

[CR7] Jarus O. 20 of the worst epidemics and pandemics in history. Live Science. 2020.

[CR8] Kang Y, Kato S (2014). An electromagnetic simulation study of the distribution of the power absorbed in evaporative humidifier elements. HVAC&R Res.

[CR9] Katara G, Hemvani N, Chitnis S, Chitnis V, Chitnis DS (2008). Surface disinfection by exposure to germicidal UV light. Indian J Med Microbiol.

[CR10] Khandpur P, Gogate PR (2016). Evaluation of ultrasound based sterilization approaches in terms of shelf life and quality parameters of fruit and vegetable juices. Ultrason Sonochem.

[CR11] Mata-Portuguez VH, Perez LS, Acosta-Gio E (2002). Sterilization of heat-resistant instruments with infrared radiation. Infect Control Hosp Epidemiol.

[CR12] McDonnell G, Russell AD (1999). Antiseptics and disinfectants: activity, action, and resistance. Clin Microbiol Rev.

[CR14] Piyasena P, Mohareb E, McKellar RC (2003). Inactivation of microbes using ultrasound: a review. Int J Food Microbiol.

[CR15] Rutala WA, Weber DJ (2016). Disinfection, sterilization, and antisepsis: an overview. Am J Infect Control.

[CR16] Summerfelt ST (2003). Ozonation and UV irradiation: an introduction and examples of current applications. Aquacult Eng.

[CR17] van Doremalen N, Bushmaker T, Morris DH, Holbrook MG, Gamble A, Williamson BN (2020). Aerosol and Surface Stability of SARS-CoV-2 as Compared with SARS-CoV-1. N Engl J Med.

[CR18] Walker RW, Markillie LM, Colotelo AH, Geist DR, Gay ME, Woodley CM (2013). Ultraviolet radiation as disinfection for fish surgical tools. Anim Biotelemetry.

[CR19] Wallace CA (2016). New developments in disinfection and sterilization. Am J Infect Control.

[CR20] Wedemeyer GA (1996) Physiology of fish in intensive culture systems. In: Conference proceedings, Springer US

[CR21] Xu D, Yang Q, Dong F, Krishnaswamy S (2018). Evaluation of surface roughness of a machined metal surface based on laser speckle pattern. J Eng.

[CR22] Yoo JH (2018). Review of disinfection and sterilization—back to the basics. Infect Chemother.

[CR13] Yoon J, Lee K, Park Y (2016) A simple and rapid method for detecting living microorganisms in food using laser speckle decorrelation. arXiv:160307343 [q-bioQM]

